# The Genetic Architecture of Chronic Mountain Sickness in Peru

**DOI:** 10.3389/fgene.2019.00690

**Published:** 2019-07-30

**Authors:** Steven Gazal, Jose R. Espinoza, Frédéric Austerlitz, Dominique Marchant, Jose Luis Macarlupu, Jorge Rodriguez, Hugo Ju-Preciado, Maria Rivera-Chira, Olivier Hermine, Fabiola Leon-Velarde, Francisco C. Villafuerte, Jean-Paul Richalet, Laurent Gouya

**Affiliations:** ^1^Department of Epidemiology, Harvard T.H. Chan School of Public Health, Boston, MA, United States; ^2^Program in Medical and Population Genetics, Broad Institute of MIT and Harvard, Cambridge, MA, United States; ^3^INSERM, Infection, Antimicrobials, Modelling, Evolution (IAME), UMR 1137, Paris, France; ^4^Plateforme de génomique constitutionnelle du GHU Nord, Assistance Publique des Hôpitaux de Paris (APHP), Hôpital Bichat, Paris, France; ^5^Laboratorio de Biotecnología Molecular-LID, Departamento de Ciencias Celulares y Moleculares, Universidad Peruana Cayetano Heredia, Lima, Perú; ^6^UMR CNRS 7206 Eco-Anthropologie et Ethnobiologie, Musée de l’Homme, Paris, France; ^7^Université Paris 13, Sorbonne Paris Cité, INSERM UMR 1272 Hypoxie et Poumon, Bobigny, France; ^8^Laboratorio de Fisiología Comparada/Fisiología de Adaptación a la Altura-LID, Departamento de Ciencias Biológicas y Fisiológicas, Universidad Peruana Cayetano Heredia, Lima, Perú; ^9^Université Paris Descartes, Institut National de la Santé et de la Recherche Médicale Unité 1163, Centre National de la Recherche Scientifique, Equipes de Recherche Labellisées 8254, Institut Imagine, Paris, France; ^10^Laboratoire d’Excellence, Globule Rouge-Excellence, Paris, France; ^11^Université Paris Diderot, INSERM U1149, Hème, fer et pathologies inflammatoires, Assistance Publique des Hôpitaux de Paris (APHP), Hôpital Louis Mourier, Paris, France

**Keywords:** chronic mountain sickness (CMS), GWAS—genome-wide association study, high altitude adaptation, natural selection, Monge’s disease

## Abstract

Chronic mountain sickness (CMS) is a pathological condition resulting from chronic exposure to high-altitude hypoxia. While its prevalence is high in native Andeans (>10%), little is known about the genetic architecture of this disease. Here, we performed the largest genome-wide association study (GWAS) of CMS (166 CMS patients and 146 controls living at 4,380 m in Peru) to detect genetic variants associated with CMS. We highlighted four new candidate loci, including the first CMS-associated variant reaching GWAS statistical significance (rs7304081; *P* = 4.58 × 10^−9^). By looking at differentially expressed genes between CMS patients and controls around these four loci, we suggested *AEBP2*, *CAST*, and *MCTP2* as candidate CMS causal genes. None of the candidate loci were under strong natural selection, consistent with the observation that CMS affects fitness mainly after the reproductive years. Overall, our results reveal new insights on the genetic architecture of CMS and do not provide evidence that CMS-associated variants are linked to a strong ongoing adaptation to high altitude.

## Introduction

Chronic mountain sickness (CMS), or Monge’s disease, is a pathological condition resulting from chronic exposure to hypoxia at high altitude. The syndrome is characterized by an excessive number of red blood cells associated with a high blood hemoglobin concentration ([Hb]), hypoxemia, and, in some cases, pulmonary hypertension. Clinical signs include headache, fatigue, sleep disturbances, dyspnea, digestive complaints, and high risk of thrombotic events. The disease may appear during early adulthood, and turn into a highly prevalent condition mainly in men over 40 years old and in post-menopausal women. The clinical status becomes progressively incapacitating with cardiovascular complications, leading to social exclusion and psychological degradation (León-Velarde et al., 2003; León-Velarde et al., 2005; Richalet et al., 2008; Espinoza et al., 2014; Villafuerte and Corante, 2016). CMS shows a unique worldwide prevalence pattern in native high-altitude dwellers. Indeed, while the prevalence of the disease is particularly high in the Peruvian Andes where more than 10% in the adult population living above 2,500 m may suffer from this condition (León-Velarde et al., 1994), the disease has not been described in the Ethiopian population living on the East African high-altitude plateau (Appenzeller et al., 2006) and is only found occasionally in Tibetans (Xing et al., 2008).

Little is known about the genetic architecture of CMS in Andean populations. We expect that this architecture is mainly driven by common genetic variants due to both the strong genetic component of [Hb] at high altitude (heritability greater than 0.8 in both Tibetan and Bolivian populations) (Beall et al., 1998) and the high prevalence (León-Velarde et al., 1994). Recent genetic studies highlighted CMS candidate genes in native Andeans (Appenzeller et al., 2006; Zhou et al., 2013; Espinoza et al., 2014; Stobdan et al., 2017), but limited sample sizes due to the challenge of research participant recruitment have prevented the investigation of CMS architecture at the genome-wide scale.

A separate question that would help to design CMS genetic studies is why the prevalence of CMS is so high in native Andeans and so low in native Tibetans and native Ethiopians. As there is strong evidence of genetic adaptation to high altitude in Tibetan and Ethiopian populations (Beall et al., 2010; Simonson et al., 2010; Yi et al., 2010; Xu et al., 2011; Alkorta-Aranburu et al., 2012; Huerta-Sánchez et al., 2013; Huerta-Sánchez et al., 2014), the first (and mainly) investigated hypothesis is that adaptation to high altitude in Andeans is still ongoing (Zhou et al., 2013; Ronen et al., 2014), and that protective genetic variants under positive selection have not yet reached their optimum frequency. In fact, archeological evidence for human presence in the Andes extends back to around 14,000 years (Rademaker et al., 2014), while the Tibetan Plateau was probably inhabited more than 30,000 years ago (Dambricourt Malassé and Gaillard, 2011). In the case of high-altitude native Tibetans, one variant of *EPAS1* (Endothelial PAS Domain Protein 1) is associated with red blood cell abundance and has an allele frequency close to 90%, against 10% in Han Chinese (Beall et al., 2010; Yi et al., 2010). A high fraction (17.8%) of Han Chinese males migrating to the Qinghai-Tibet plateau develop CMS (Jiang et al., 2014); however, CMS is much less frequent in native Tibetans (Mejia et al., 2005), suggesting a link between adaptation to high altitude and the prevalence of CMS. Under this first hypothesis, we thus assume a strong effect of genetic variants on CMS phenotype; several studies have proposed CMS candidate genes by contrasting allele frequencies in CMS and controls in regions under positive selection (Zhou et al., 2013; Stobdan et al., 2017). A second and non-exclusive hypothesis would be that Andeans were on their way to be adapted to high altitude, but recent (400 years) admixture with European populations re-introgressed in the population lowlander genetic variants that are non-adapted to high altitude, and thus could increase one’s risk for CMS. Higher CMS prevalence in men of European descent in the Andes reinforces this second hypothesis (Monge, 1943; Ergueta et al., 1971; Mejia et al., 2005). Finally, a third hypothesis would be that high CMS prevalence in Andes is a consequence of the different process of functional adaptation observed in Tibetans and Andeans (Beall, 2007), as the Tibetan process of high-altitude adaptation, limiting [Hb] increase through mainly an adaptation of the hypoxia-inducible factor (HIF) pathway, is also protective of CMS, while the Andean process is not. Indeed, CMS is mainly a late-onset disease where most of the known deleterious conditions occur after the reproductive period (20–25 years is usually considered as the peak of the reproductive period) and therefore may be under moderate selection. We thus might not expect a strong link between variants that are (or have been) under adaptation and the ones that increase one’s risk for CMS.

We cannot discard the hypothesis that physiological acquired factors, rather than genetic variants, are relevant to the development of CMS. In fact, CMS patients have an slightly increased body mass index, more frequent sleep apneas, and alteration in chemosensitivity; all of these factors lead to decreased ventilation, hypoxemia, and increased erythropoiesis (León-Velarde et al., 2005; Richalet et al., 2008; Villafuerte and Corante, 2016). Polycythemia, by itself, may also reduce ventilation, leading to a vicious circle reinforcing polycythemia (Villafuerte et al., 2007).

To understand the genetic architecture of CMS in the Andes and the population genetic forces shaping it, we conducted the largest genome-wide association study (GWAS) of this disease by successfully genotyping on extremely dense single nucleotide polymorphism (SNP) chips 166 CMS patients and 146 healthy subjects living at Cerro de Pasco in Peru (altitude: 4,380 m) where CMS prevalence is around 15% (León-Velarde et al., 1994). First, we discovered the first genome-wide significant variant of CMS (rs7304081) and identified three other candidate variants (rs75810402, rs7832232, and rs7168430). We measured gene expression in peripheral blood under hypoxia condition for 71 unrelated patients to suggest potential causal genes around our GWAS candidate loci and to bring additional evidence for some previously reported CMS candidate genes. Finally, we performed various tests of selection at the whole-genome scale and an admixture mapping and found no signal shared with the association analysis, favoring our third hypothesis that CMS risk variants are not under current adaptation.

## Material and Methods

### Patients and Controls

The study population was composed of 387 residents of the city of Cerro de Pasco (4,380 m, Peru). They were all high-altitude native of Quechua ancestry, as is most of the population of Cerro de Pasco. Only male individuals with residence longer than 6 months in Cerro de Pasco were recruited in the study. Individuals were not enrolled in the study if they had any other chronic disease or smoking habit (≥5 cigarettes per day). The study was approved by the Institutional Ethics Committee of Universidad Peruana Cayetano Heredia. All participants were enrolled in the study after signing an informed consent.

### Clinical and Physiological Profile of the Population

A complete clinical evaluation was made, and CMS clinical score calculated. CMS score is based on the following symptoms: breathlessness, sleep disturbance, cyanosis, paresthesia, headache, and tinnitus (León-Velarde et al., 2005). The score included hematocrit ≥ 63% cutoff and clinical symptoms (León-Velarde et al., 2005). In the subset of patients that passed quality control (i.e., patients with no duplicated genetic data and with an appropriate genotype call rate; see below), 166 were CMS patients (46.8 ± 13.4 years old) and 146 were healthy controls of similar age (43.0 ± 13.1 years old). None of the subjects had been traveling for more than 1 month at low altitude in the preceding 6 months, and none were recently working in mining facilities. The evaluation of pulmonary function was performed before inclusion to exclude subjects with pulmonary diseases. Forced vital capacity (FVC) and forced expired volume in one second (FEV_1_) were measured by spirometry (Microloop Spirometer, MicroMedical Ltd., Rochester, UK). All values were within normal limits corrected for age, sex, and height. Blood was drawn from an antecubital vein. Hematocrit was evaluated by microcentrifugation (Microcentrifuge IEC, Thermo Electron, Waltham, MA), and pulse O_2_ saturation was measured by transcutaneous oximetry (Nellcor N-595, Nellcor, Pleasanton, CA, USA). 12-lead electrocardiography (EKG) was performed, and usual markers of coronary disease, conduction, or rhythm disorders were looked for. Indirect signs of pulmonary hypertension were detected through right ventricular hypertrophy [RVH: right axis deviation ≥120°, tall R wave in V1 plus persistent precordial S waves (R-V1 + S − V5 > 10.5 mm)]. Systemic arterial pressure was measured by sphygmomanometry after a 15-min rest in supine position. A quality of life score, adapted and validated to Spanish language, assessed through a form, was completed by the patients under the supervision of a physician (Mezzich et al., 2000).

### DNA Extraction, Genotyping, and Quality Control

A blood sample was drawn from 387 participants. Frozen blood tubes were delivered to the laboratory. Leukocytes DNA was extracted by a salting out procedure (Sambrook et al., 1989). DNA was quantified and qualified with NanoDrop™ 2000 (260/280 ≥ 2.0; 260/230 ≥ 1.8) and stored at −20°C until use. All individuals were genotyped on Affymetrix array (4,363,966 SNPs). We first removed duplicated individuals and kept individuals with a genotype call rate ≥95%. Then, we removed SNPs with missingness greater than 5%. We restricted to SNPs on autosomes and the X chromosome with a minor allele frequency (MAF) >5%. These quality control steps resulted in a sample of 312 individuals (166 CMS patients and 146 controls) genotyped on 1,288,119 SNPs; most of the removed samples were due to duplicates, and most of the removed SNPs were due to our high MAF threshold. For further analyses requiring a set of unrelated individuals, we created a set of 267 unrelated individuals (143 CMS patients and 124 controls) with a genomic kinship coefficient lower than 1/16. Kinship coefficients were estimated using PLINK (Purcell et al., 2007) using option –genome on a pruned dataset [PLINK option –indep-pairwise 50 5 0.50, as recommended by Anderson et al. (Anderson et al., 2010)].

### Population Structure

To study the structure of our sample, we used both the Human Genome Diversity (HGDP-CEPH) panel (Cann et al., 2002) and the 1000 genomes project (1000G) panel (1000 Genomes Project Consortium, 2010; 1000 Genomes Project Consortium, 2012; Auton et al., 2015). The HGDP-CEPH panel genotyped 942 unrelated individuals from 52 populations from seven geographic regions (including Native American populations). Phase 3 of 1000G panel sequenced 2,504 unrelated individuals from 26 populations from five geographic regions (including European populations and a Peruvian population from Lima). We merged our initial dataset and these two reference panels by selecting common markers in autosomes with the same alleles in each dataset. We then kept markers that were polymorphic (MAF > 1% in each HGDP-CEPH region, and MAF >1% in each 1000G region) and that respected Hardy–Weinberg equilibrium (*P* value > 10^−5^ in our dataset, and in each HGDP-CEPH and 1000G region). After these steps, 133,649 SNPs remained. This set of SNPs was only used for population structure analyses and admixture analyses (see below).

Principal component analysis (PCA) was performed using PLINK. We first pruned the previous dataset to remove sites in linkage disequilibrium (still using PLINK option –indep-pairwise 50 5 0.50) and projected our 312 individuals on the 3,446 individuals of HGDP-CEPH and 1000G panels.

The genome-wide proportion of European and Native American ancestries of individuals from our dataset was estimated with the software Admixture (Alexander et al., 2009). To construct a Native American reference ancestral population, we first run Admixture unsupervised clustering algorithm with the default options and *K* = 2 ancestral population on the 64 Americans individuals of HGDP-CEPH panel and the 503 Europeans of 1000G panel (**Figure S1A**). The 21 American individuals that have been inferred as entirely coming from the first ancestral population were included in the Native American reference ancestral population (these individuals come from Karitiana and Surui populations). The 21 European individuals having the highest proportion of the genome coming from the second ancestral population were included in the European reference ancestral population in order to have two reference ancestral populations of the same size. The genome-wide proportion of European and Native American ancestries of our individuals was then estimated using Admixture supervised clustering algorithm and these two reference ancestral populations. Each Admixture analysis was performed after a pruning of the data as recommended by Admixture authors (using PLINK option –indep-pairwise 50 5 0.10). We note that because we used a supervised analysis, admixture results are not influenced by the relatedness in the sample. The genome-wide proportion of European ancestry was compared between unrelated CMS patients and controls using a logistic regression with age and batch effect as covariates. We also performed non-parametric tests but found no statistical significance between CMS patients and controls (not shown). We note that we did not investigate African admixture due to negligible proportion of African ancestry in our samples (not shown); we also note that in an Admixture unsupervised analysis of our dataset, *K* = 2 reached the best predictive accuracy in cross validation analysis among *K* = 2, 3, and 4 (0.594, 0.604, and 0.614, respectively).

We did not investigate Denisovan admixture, for which an *EPAS1* haplotype has been shown to be associated with high-altitude adaptation in Tibet (Huerta-Sánchez et al., 2014), due to the overall limited proportion of Denisovan admixture in Native Americans (Sankararaman et al., 2016) and to the absence of this haplotype in our sample after performing imputation. Indeed, only one individual carried the Denisovan variant of rs115321619, and the other four SNPS (rs73926263, rs73926264, rs73926265, and rs55981512) were monomorphic for the non-Denisovan variant in our sample [these five variants have been imputed using all sequenced individuals from Phase 3 of 1000G panel and by following IMPUTE2 (Howie et al., 2009; Howie et al., 2012) best practices by pre-phasing the data using SHAPEIT2 (Delaneau et al., 2013)].

### Association Analysis

We tested, under an additive model, the association between each of the 1,288,119 genotyped SNP and CMS. A preliminary association power analysis was performed using the QUANTO software (Gauderman and Morrison, 2009). Specifically, we computed for different allele frequencies (ranging from 5% to 50% with a step of 5%) and different significance thresholds (5 × 10^−8^, 10^−5^, and 10^−4^). We computed the genetic effect that we can detect with a power of 90% and the power to detect a genetic effect of 2.0, 2.5, and 3.0. To take into account the structure and the relatedness of our sample, we performed an association analysis using the standard linear mixed model implemented in GEMMA software (Zhou and Stephens, 2012). Relationships between individuals were estimated using a centered relatedness matrix. {We note that we did not computed individual allele frequencies based on individual European and Native American admixture of each individual (as performed in Moltke et al., 2014) due to i) our relative small sample size of Native American individuals (*N* = 21) and ii) the low level of European admixture in our dataset (11.30 ± 6.15%; median = 9.54). Genotypes, age, and the batch effect were modeled as fixed effects, while relatedness was treated as a random effect. We did not use body mass index (BMI), right ventricular hypertrophy (RVH), and oxygen saturation (SaO_2_) as covariates (also associated with CMS, see Results), as there is no evidence that they are cause or consequence of the disease (instead, we performed association analyses for every candidate SNPs for all these phenotypes in order to better understand the SNP causality with these three phenotypes). Significance of each SNP was determined through a likelihood ratio test. We defined SNPs with a *P* value < 10^−5^ as candidate. When multiple candidate SNPs were in the same region, we only reported the top SNP with the smallest *P* value. We performed conditional analyses to verify that the significance of the surrounding candidate SNPs is due to linkage disequilibrium with the top SNP. Regression coefficients from the linear mixed model (and corresponding 95% confidence intervals) were converted to odds ratio for easier interpretation as proposed by Lloyd-Jones et al. (Lloyd-Jones et al., 2018). To compute the proportion of the variance explained by the most associated SNP (rs7304081), we performed a logistic regression (adjusted on age and batch effect) on the subset of 267 unrelated individuals, computed the proportion of variance explained on the observed scale as its chi square statistics divided by the sample size, and converted this number from the observed scale to the liability scale using the formula of Lee et al. (Lee et al., 2011). To investigate potential higher signal around candidate regions, we imputed variants using all sequenced individuals from Phase 3 of 1000G panel (see previous paragraph). As imputation results have to be read carefully (Cerro de Pasco population is not represented in the imputation reference panel and/or might have experienced recent adaptation), we did not report them in the main version of the manuscript.

### Gene Expression Analyses

Gene expression was measured for candidate genes in a subset of 71 unrelated individuals (30 CMS patients and 41 controls) using RNA extracted from whole blood under hypoxia condition (PAXgen; Becton Dickinson). We manually selected candidate gene around the four candidate regions (based on known function of genes and by giving a higher emphasis to the region containing the genome-wide significant SNP). We also selected 10 additional genes reported in other studies as CMS candidate genes or genes being under adaptation to high altitude. Blood samples were withdrawn in PAXgen tubes and immediately frozen until analyses. After extraction, an RNA quality control was performed on Fragment Analyzer (AATI) with the RNA kit (DNF-471) to check the integrity of the RNA profile and to estimate the RNA concentration. A Reverse Transcription reaction was performed for each sample in 20 µl, according the conditions of the High Fidelity Reverse Transcription kit (Applied Biosystems). qPCRs were performed on the Biomark (Fluidigm) in a microfluidic multiplex 96.96 dynamic array chip according to the Fluidigm Advanced Development Protocol with EvaGreen (PN 100 – 1208 B1). One chip was used to quantify all transcripts. A 14-cycle preamplification reaction was performed for each sample in 10 µl by pooling 48 primer pairs (final concentration, 50 nM), 3.3 µl cDNA, and 5 µl 2X PreAmp Master Mix (Applied Biosystems) according to Applied Biosystems conditions. For each individual assay, 5 µl 10X Assay Mix containing 9 µM forward primer, 9 µM reverse primer, and 1X Assay Loading Reagent was loaded into one of the Assay Inlets on the chip. The following solution (5 µl) was loaded in sample inlets: 1.25 µl Preamplified sample previously diluted in low TE Buffer, 2.5 µl 2X Taqman Gene Expression Master Mix (Applied Biosystems), 0.25 µl 20X DNA Binding Dye Sample Loading Reagent (Fluidigm, PN 100-0388), 0.25 µl 20X EvaGreen (Biotium), and 0.75 µl low TE Buffer. The Biomark’s default cycling program was used to amplify fragments. All experiments were done in four replicates. CT values were obtained using BioMark Gene Expression Data Analysis version 3.0.2 according to Fluidigm’s recommendations for EvaGreen Gene Expression. Gene expression was normalized using *HMBS* (hydroxymethylbilane synthase) and *GAPDH* (glyceraldehyde-3-phosphate dehydrogenase) as reference. Expression levels were log transformed. We compared the expression between CMS patients and controls using a logistic regression with age as a covariate.

### Detection of Recent Positive Selection

Selection tests were performed on the subset of 124 unrelated controls. For all SNPs with a MAF above 5% in the whole sample, we first computed, using the selscan program (Szpiech and Hernandez, 2014), the iHS (Voight et al., 2006) and nSL (Ferrer-Admetlla et al., 2014) indices, which are haplotypic tests that take high values in the case of recent positive selection on a given SNP. For each iHS (resp. nSL) statistic, a *P* value was computed as the proportion of SNPs in the sample having a lower or equal index.

### Pathway Analysis

To assess the overall evidence of association between hypoxia-inducible factor (HIF) pathway and CMS and natural selection, we retained 345 autosomal genes in the nine Gene Ontology (GO) categories retained by Simonson et al. (Simonson et al., 2010): detection of oxygen (GO:0003032), nitric oxide metabolic process (GO:0046209), oxygen sensor activity (GO:0019826), oxygen binding (GO:0019825), oxygen transport (GO:0015671), oxygen carrier activity (GO:0005344), response to hypoxia (GO:0001666), response to oxygen levels (GO:0070482), and vasodilation (GO:0042311). For each gene and each study (i.e., CMS GWAS, iHS, and nSL), we retained the minimal *P* value inside the gene body. We also replicated our analyses by retaining the minimal *P* value in +/−20 kb and +/−50 kb windows around the genes to include regulatory variants. We next computed association enrichment using two complementary gene set analyses. First, we executed an over-representation analysis that calculates the proportion of genes in the pathway having a *P* value less than 0.05 (results were also replicated using a 0.01 threshold). This approach has been widely and successfully used for pathway analysis; see Holmans (Holmans, 2010) for a review. Second, to take into account all the genes *P* values, we performed the original Fisher product method (Fischer, 1932), which has been demonstrated to be powerful under different simulation scenarios (Fridley et al., 2010). *P* values for these methods were computed using 5,000 permutations that randomly shift genome annotations (Cabrera et al., 2012). We note that the aim of this approach is to compare SNPs in the HIF-pathway to other SNPs with similar LD structure and clustering; the aim of this approach is not to compare HIF-pathway to other pathways.

### Admixture Mapping

In order to investigate the role of European genomes on our phenotypes, we performed admixture mapping to test if chromosomal segments inherited from European populations were associated with CMS. Inference of chromosomal segments ancestry was performed using RFMix (Maples et al., 2013) with two reference ancestral haplotype populations, obtained by phasing Americans individuals of the HGDP-CEPH and Europeans of 1000G panels with SHAPEIT2 (Delaneau et al., 2013). Selecting the 21 Native American and 21 European samples used previously overestimated European local admixture estimations (data not shown). We thus used haplotypes from all the 64 American individuals of HGDP-CEPH panel and the 503 Europeans of 1000G panel in the reference ancestral haplotype populations. Averaging the European admixture proportion estimated at each marker by RFMix gave similar values to the genome-wide European admixture proportion estimated previously by the Admixture software (correlation = 0.98; **Figure S1B**), validating our approach.

For each individual, and for each of the 133,649 SNPs common to the HGDP-CEPH and 1000G data, we obtained an average number of alleles of European origin (between 0 and 2) deduced from forward-backward RFMix output probabilities. GEMMA was then used to test the association of these probabilities with the different phenotypes, by using the same covariates as in the association analyses. Finally, to merge admixture-mapping results with association results, we interpolated linearly –log10 of the *P* values for SNPs that were not present in the 133,649 SNPs used for the analysis.

## Results

### Phenotypic Description of the Sample

The study population was composed of 387 residents of the city of Cerro de Pasco (4,380 m, Peru). After quality control, we retained 166 CMS patients and 146 controls (312 total genotyped individuals). Clinical and physiological data of these patients are presented in **Table 1**. CMS patients have similar age as controls (see also **Figure S2**). CMS cases showed higher body weight and body mass index (**Figure S3A**). By construction, hematocrit and CMS clinical score are significantly higher within cases. Moreover, cases show lower resting SaO_2_ (**Figure S3B**) and higher heart rate, but no significantly different systemic arterial pressures. Cases frequently show electrocardiographic signs of right ventricular hypertrophy. Also, cases showed slightly less frequent trips to sea level. The overall Quality of Life score as well as the physical and psychological/emotional well-being items are not significantly different between cases and controls.

**Table 1:** Clinical and physiological variables in chronic mountain sickness patients (CMS) and controls (CTRL).

Variable	*N* (CMS/CTRL)	CMS	CTRL	*P*-value
Age (years)	166/146	46.8 ± 13.4	43.0 ± 13.1	NS
Body weight (kg)	166/146	68.8 ± 9.2	64.9 ± 8.7	0.001
Height (cm)	166/146	162.6 ± 5.9	163.0 ± 5.9	NS
Body mass index (kg/m2)	166/146	26.1 ± 3.3	24.4 ± 2.7	<0.001
Hematocrit (%)	166/146	67.2 ± 3.8	52.0 ± 3.2	<0.001
SpO_2_ (%)	166/146	85.3 ± 5.2	89.7 ± 4.8	<0.001
Heart rate (b/min)	165/144	72.1 ± 11.3	67.6 ± 9.2	<0.001
Systolic BP (mmHg)	164/144	116.3 ± 15.2	115.4 ± 13.6	NS
Diastolic BP (mmHg)	164/144	76.9 ± 10.3	76.0 ± 10.1	NS
CMS clinical score	164/144	6.8 ± 3.6	2.2 ± 2.1	<0.001
RVH	166/146	120 (72%)	61 (42%)	<0.001
Smoking habit (0–4 cig./day)	141/100	26 (18%)	21 (21%)	NS
Alcohol use	141/101	64 (45%)	52 (51%)	NS
Birth at high altitude	165/146	157 (95%)	136 (93%)	NS
Parents from high altitude	166/146	159 (96%)	138 (95%)	NS
Frequent trips to sea level	140/101	77 (55%)	72 (71%)	0.009
Past history of work in mine	166/146	23 (14%)	12 (8%)	NS
Physical well being	94/56	6.2 ± 1.9	6.7 ± 2.0	NS
Psychological/emotional well being	94/55	6.6 ± 2.0	7.1 ± 1.8	NS
Overall Quality of Life score	94/55	68.8 ± 15.1	70.5 ± 12.7	NS

SpO_2_, arterial oxygen saturation; BP, blood pressure; RVH, right ventricular hypertrophy. Results presented are mean ± standard deviation or n (%). P values were computed using logistic regressions on 267 unrelated individuals (143 CMS and 124 controls), adjusting for batch effect for quantitative and qualitative variables. NS, non-significant.

### Genetic Population Structure

**Figure 1:**
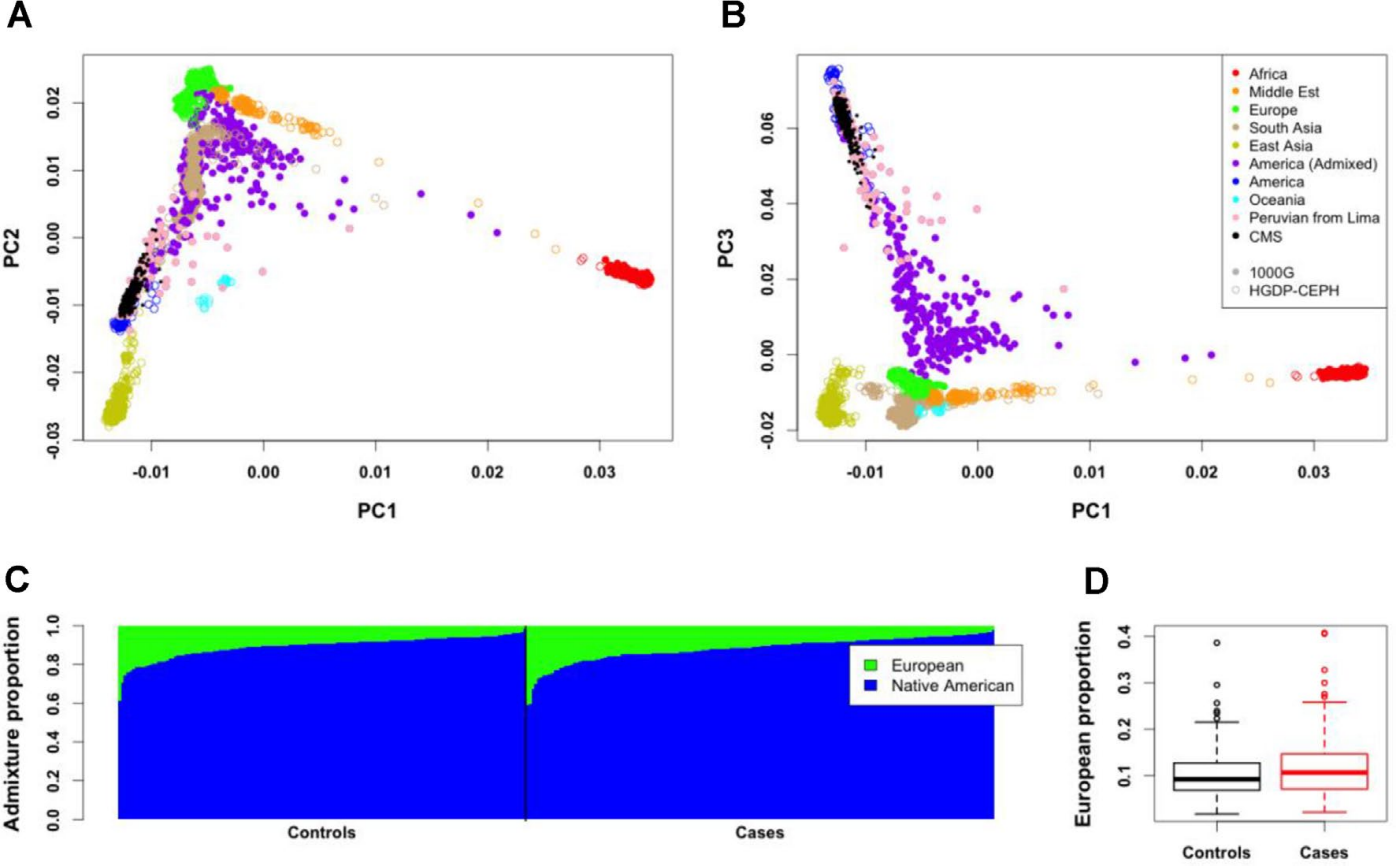
Genome-wide structure of Cerro de Pasco population. **(A,B)** Projection of the 312 individuals (small black points) on Human Genome Diversity (HGDP-CEPH) (color open points) and 1000G (color close points) panels. Peruvian from Lima (PEL) of the 1000G panel are represented with pink points. **(C)** Admixture proportions of European and Native American ancestries for the 312 individuals. These proportions were estimated from 21 Native American of HGDP-CEPH panel and 21 European from 1000G panel. Each vertical line represents an individual. **(D)** Mean European admixture proportions (and quantiles) in unrelated CMS patients and controls.

To study the proportion of European genome present in our data, we first projected our 312 individuals with Quechua ancestry on the HGDP-CEPH (942 unrelated individuals from 52 populations, including European and Native American populations) and 1000 Genomes (1000G; 2,504 unrelated individuals from 26 populations, including European populations and a Peruvian population from Lima) panels (**Figures 1A, B**). PCA results show that our individuals are close to the cluster of Native Americans of HGDP-CEPH panel and to the Peruvian from Lima of 1000G, but spreading toward the European cluster. We then computed the proportion of European ancestries for all individuals by using a Native American and European sample as reference ancestry populations (**Figure 1C**; see Methods). Around half of the individuals (160 individuals) have less than 10% European ancestry, while 20% showed more than 15% European ancestry (58 individuals, including 28 individuals with more than 20%). The proportion of European admixture in unrelated CMS cases (11.59 ± 6.37%; median = 10.25) tended to be higher than in unrelated controls (10.96 ± 5.89%; median = 9.09); however, this difference was not significant (*P* = 0.73; **Figure 1D**).

### Genome-Wide Association Study

**Figure 2:**
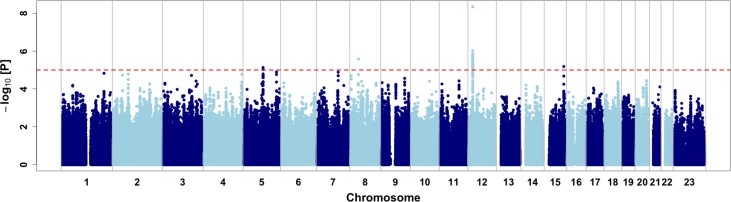
Genome-wide association results. Dashed red line represents the discovery threshold for selection of candidate SNPs (*P* < 10^−5^).

We then performed a genome-wide association study on the CMS status (**Figure 2**, **Table 2**, and **Table S1**). Relatedness, inbreeding, and population structure were taken into account through a linear mixed model, with age and batch effect used as a covariate with a fixed effect. A preliminary power study showed that our sample size was underpowered at the conventional genome-wide significance threshold of 5 × 10^−8^ but had a reasonable power (≥60%) to detect very common variants (MAF ≥20%) with odd ratios of 2.5 when considering a discovery threshold of 10^−5^ (**Figure S4**). We note that this effect is on the order of magnitude of the strongest allele frequency differences observed in samples with 7K Hans and 3K Tibetans, including the variants of *EPAS1* and *EGLN1* (Egl-9 Family Hypoxia Inducible Factor 1) genes (Yang et al., 2017). Nevertheless, we found one genome-wide significant SNP on chromosome 12 (rs7304081, OR = 2.52, 95% CI [1.85; 3.48], *P* = 4.58 × 10^−9^). We note the large effect size of this SNP (OR = 2.52, significant association consistent with our power study) especially due to its high frequency in the population (57% in CMS and 34% in controls), which explains 13% of the trait variance. We also found three other independent candidate loci with *P* < 10^−5^ on chromosomes 5 (rs75810402, OR = 0.28, 95% CI [0.13;0.50], *P* = 7.37 × 10^−6^), 8 (rs7832232, OR = 2.16, 95% CI [1.56;3.04], *P* = 2.63 × 10^−6^), and 15 (rs7168430, OR = 2.23, 95% CI [1.57;3.24], *P* = 6.48 × 10^−6^). Imputation around these loci did not reveal new significant information (**Figures S5–S8**), and conditional analyses did not reveal independent signal in these four candidate regions (Figure S9).

**Table 2:** Characteristics of main loci associated to chronic mountain sickness (CMS) disease.

Variant	Chr	Position (hg19)	Alleles^1^	OR [95% CI]	*P* GWAS	Nearest genes^2^	Candidate gene^3^	*f* CMS	*f* CTRL	*f* PEL	*P* iHS	*P* NSL
rs75810402	5	96,747,224	**G**/A	3.57 [2.00; 7.69]	7.37 × 10^−6^	*RIOK2, LINC01340*	CAST	0.96	0.87	0.96	0.33	0.29
rs7832232	8	38,469,303	**G**/A	2.16 [1.56; 3.04]	2.63 × 10^−6^	*RNF5P1, TACC1*	–	0.42	0.24	0.34	0.96	0.85
rs7304081	12	19,561,543	**A**/C	2.52 [1.85; 3.48]	4.58 × 10^−9^	*PLEKHA5, AEBP2*	*AEBP2*	0.57	0.34	0.44	0.99	0.94
rs7168430	15	94,349,452	G/**A**	2.23 [1.57; 3.24]	6.48 × 10^−6^	*RGM1, MCTP2*	*MCTP2*	0.38	0.22	0.32	0.91	0.75

Chr, chromosome; OR, odd ratio of the minor allele; CI, confidence interval; P, P value; f, allele frequency of the risk allele; CMS, chronic mountain sickness patients; CTRL, controls; PEL, Peruvian from Lima; RIOK2, serine/threonine-protein kinase 2; LINC01340, long intergenic non-protein coding RNA 1340; CAST, calpastatin; RNF5P1, ring finger protein 5 pseudogene 1; TACC1, transforming acidic coiled-coil containing protein 1; PLEKHA5, pleckstrin homology domain containing A5; AEBP2, adipocyte enhancer-binding protein; RGMA, repulsive guidance molecule BMP co-receptor A; MCTP2, multiple C2 and transmembrane domain containing 2.

^1^risk/protective allele in our sample, ancestral allele in bold. ^2^the previous and following genes around the variant (none of the variants fall inside a gene); ^3^ candidate gene based on expression analyses.

Next, we also investigated if these four variants were associated with body mass index (BMI), right ventricular hypertrophy (RVH), and pulse oxygen saturation (SpO_2_), as these phenotypes are risk factor of CMS (**Table 1**). For rs7304081 and rs7832232, the CMS risk alleles were associated with increased BMI (*P* = 8.86 × 10^−3^ and *P* = 7.78 × 10^−3^, respectively); for rs7832232, the CMS risk allele was associated with RVH risk (*P* = 0.025); and for rs75810402 and rs7168430, the CMS risk alleles were associated with decreased SpO_2_ (*P* = 9.15 × 10^−3^ and *P* = 3.25 × 10^−4^, respectively). As a consequence, adjusting for BMI, RVH, and SpO_2_ decreased the significance of the CMS association with all the candidates (*P* between 5.48 × 10^−7^ and 4.68 × 10^−3^), except for rs7832232. All association results are presented in **Table S2**. We note that we did not observe more significant results when looking at pleiotropic effects in imputed SNPs in regions surrounding these four variants (**Figures S10–S12**). Finally, the three candidate variants rs75810402, rs7832232, and rs7304081, BMI, and SpO_2_ are still significant after conditioning on each other (*P* < 0.05/6; rs7168430 is not significant due to its high association with SpO_2_; **Table S3**), indicating that each provides independent information relating to CMS.

**Figure 3:**
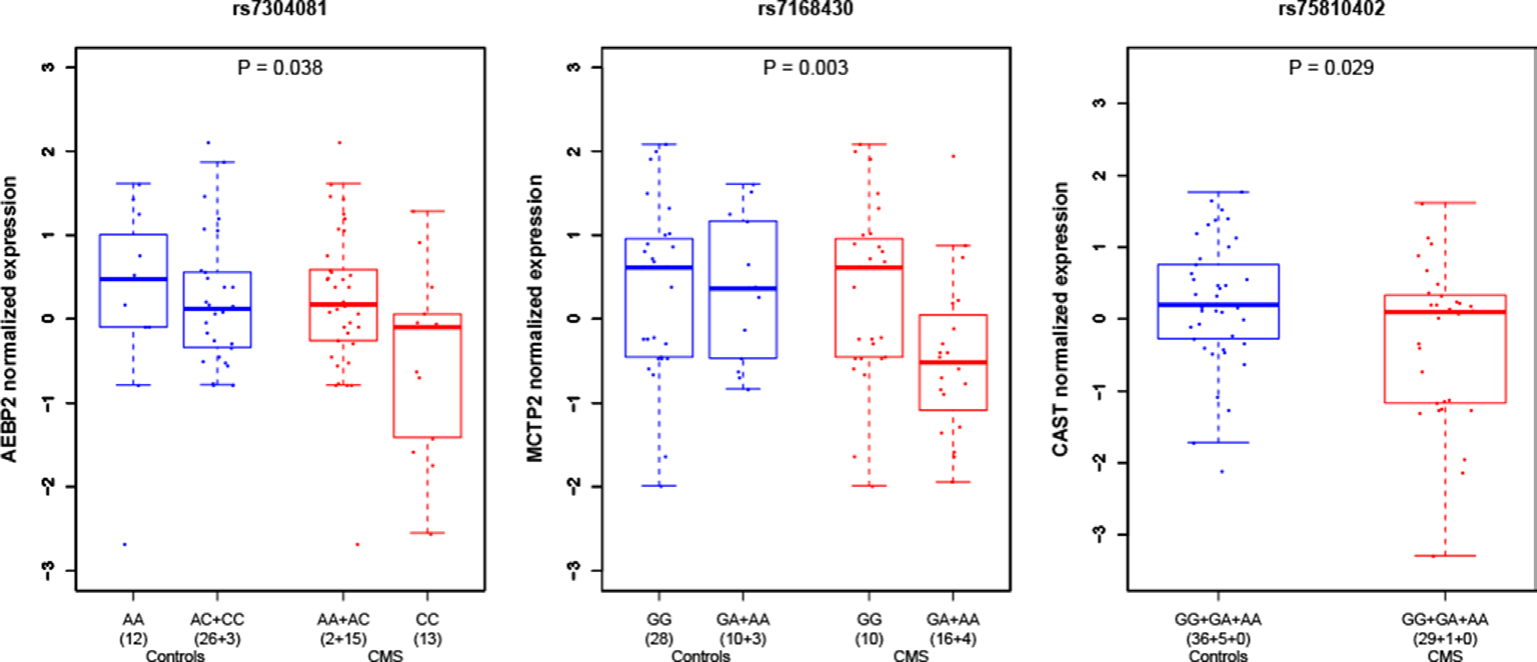
Gene expression in peripheral blood under hypoxia condition in CMS cases and controls around candidate loci. *P* values indicate the difference of level of expression in CMS cases (red) and controls (blue). Expression values were clustered into genotypes for representation purposes; note that for rs7304081, heterozygous genotypes AC were clustered with CC in controls and AA in cases.

We performed RNA quantification extracted from whole blood for seven manually selected candidate genes around the genome-wide significant SNP of chromosome 12 (rs7304081) on 71 unrelated individuals of our study (30 cases and 41 controls). The only gene with a significant difference of RNA expression between cases and controls (at the level *P* < 0.05, no correction for multiple testing) was the closest gene *AEBP2* (adipocyte enhancer-binding protein; *P* = 0.038), with lower expression levels in cases than in controls (**Figure 3** and **Table S4**). Within the 71 individuals, we also found that the number of risk variants was associated with a decrease in RNA expression (*P* = 0.042). We also performed RNA quantification for manually selected candidate genes around other candidate loci and found a significantly different expression in CMS cases and controls for genes *MCTP2* (multiple C2 and transmembrane domain containing; close to rs7168430; lower expression levels in cases than in controls; *P* = 0.003) and *CAST* (calpastatin; close to rs75810402; lower expression levels in cases than in controls; *P* = 0.029).

Finally, we performed expression analyses on 10 additional genes reported in other studies as CMS candidate genes or genes being under adaptation to high altitude [as reported in studies (Appenzeller et al., 2006; Xing et al., 2008; Bigham et al., 2010; Simonson et al., 2010; Peng et al., 2011; Xu et al., 2011; Buroker et al., 2012; Zhou et al., 2013; Espinoza et al., 2014; Udpa et al., 2014); see **Tables S4** and **S5**]. We found significantly different expression (*P* < 0.05) in CMS for three CMS candidate genes: *SENP1* (sentrin-specific protease 1) (Zhou et al., 2013), *ATM* (ATM serine/threonine kinase) (Appenzeller et al., 2006), and *VEGFA* (vascular endothelial growth factor A) (Buroker et al., 2012; Espinoza et al., 2014) (**Figure S13**). We did not observe significantly different expression for genes described as being under adaptation to high altitude (**Table S4**), even if we observed non-significant but small *P* values for EPAS1 (Simonson et al., 2010; Yi et al., 2010; Peng et al., 2011; Xu et al., 2011) and EGLN1 (Bigham et al., 2010; Peng et al., 2011; Xu et al., 2011) genes (*P* = 0.06 for both genes).

### Relationship Between GWAS, Selection Tests, and Admixture Mapping

**Figure 4:**
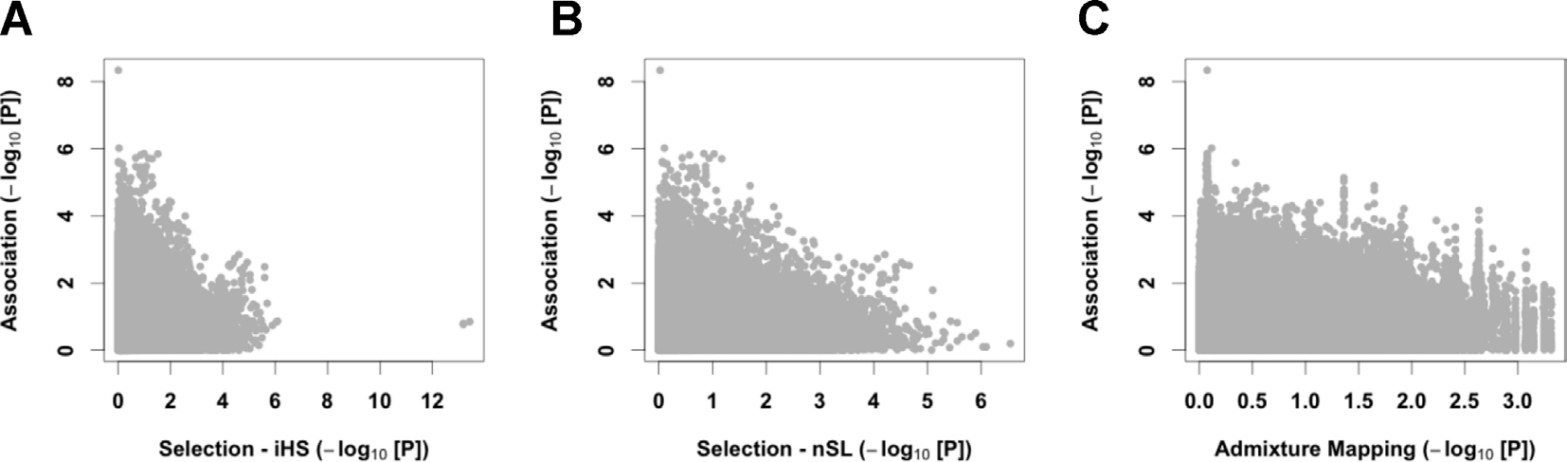
Cross-results of association signals with selection scans and admixture mapping. We compared GWAS signals with the iHS (**A**) and nSL (**B**) tests of recent positive selection and European admixture mapping (**C**).

We then merged our association results with signals of natural selection. We first computed on 124 unrelated controls two haplotypic tests of recent positive selection, iHS (Voight et al., 2006) and nSL (Ferrer-Admetlla et al., 2014). We observed strong and consistent genome-wide iHS and nSL selection signals (**Figure S14**). Interestingly, we found enrichment of nSL statistics within genes of the hypoxia-inducible factor (HIF) pathway (345 autosomal genes, *P* between 0.002 and 0.022 using different gene-set analyses; see **Table S6**), consistent with adaptation to high altitude acting on the genes of this pathway (Bigham et al., 2010; Simonson et al., 2010; Foll et al., 2014). Despite this signal of adaptation to high altitude, no strong iHS and nSL signals were shared with the association analysis (**Figures 4A, B** and **Table S1**), and we found no enrichment of GWAS signal within the HIF pathway (**Table S6**). Our four candidate variants also did not present strong iHS and nSL signals in their surrounding regions (**Table 2** and **Figure S15**). However, we note that for three out of these four variants, the risk alleles are the ancestral alleles, and all risk variants have a lower frequency in controls than in 1000G Peruvian population from Lima, but a higher frequency in CMS patients than in this Peruvian population (**Table 2**). If we assume that 1000G Peruvians are lowlanders with low Quechan ancestry, these observations suggest some selective pressure on these variants, but no strong ongoing adaptation as previously hypothesized (see Discussion).

Since we observed a tendency to have a higher proportion of European admixture in our cases (**Figure 1D**), we also performed admixture mapping to test if regions of the genome have an excess of European genome in cases. Here again, we found no strong signal shared with the association analysis (**Figure 4C** and **Table S1**).

Finally, we took advantage of our large GWAS dataset to investigate association and selection results around 21 CMS candidate genes or genes under adaptation to high altitude [as reported in studies (Appenzeller et al., 2006; Xing et al., 2008; Bigham et al., 2010; Simonson et al., 2010; Peng et al., 2011; Xu et al., 2011; Buroker et al., 2012; Zhou et al., 2013; Eichstaedt et al., 2014; Espinoza et al., 2014; Udpa et al., 2014; Crawford et al., 2017; Stobdan et al., 2017); see **Table S5** and **Figure S16**]. We confirmed strong signal of recent positive selection (*P* < 10^−5^) in a 1-Mb window around the genes *SENP1* (Zhou et al., 2013), *ANP32D* (acidic nuclear phosphoprotein 32 family member D) (Zhou et al., 2013), and *PYGM* (glycogen phosphorylase muscle associated) (Crawford et al., 2017) and moderate signal (*P* < 10^−3^) around the genes *TBX5* (T-Box 5) and *SH2B1* (Src homology 2 B adaptor protein 1) (Crawford et al., 2017). However, none of these signals contained evidence of association with CMS (**Figure S16**). Finally, we did not replicate the selection signal around the *EGLN1* gene (Bigham et al., 2010; Peng et al., 2011; Xu et al., 2011), considered as being the main gene under positive selection in both Tibetan and Andean native populations, as well as selection and association signals around *SGK3* (serum/glucocorticoid regulated kinase family member 3), *COPS5* (COP9 signalosome subunit 5), *PRDM1 (PR/SET domain 1)*, *IFT122* (intraflagellar transport 122) (Stobdan et al., 2017), *BRINP3* (bone morphogenetic protein/retinoic acid inducible neural-specific 3), and *NOS2* (nitric oxide synthase 2) (Crawford et al., 2017).

## Discussion

In this study, we performed the largest GWAS on CMS. We highlighted four new candidate loci, including the first CMS-associated variant reaching GWAS statistical significance (rs7304081; *P* = 4.58 × 10^−9^). By looking at differentially expressed genes between CMS patients and controls, we suggested *AEBP2*, *CAST*, and *MCTP2* as potential causal genes, and bring additional evidence for CMS candidate genes, including HIF pathway genes *SENP1* (Zhou et al., 2013) and *VEGFA* (Buroker et al., 2012; Espinoza et al., 2014), as well as *ATM* (Appenzeller et al., 2006). None of our candidate loci were under strong natural selection, consistent with the observation that CMS affects fitness mainly after the reproductive years (León-Velarde et al., 1993); in our population of CMS patients, only 13% were below 30 years old (**Figure S2**). Genes from the HIF-pathway were enriched for signals of natural selection but not in CMS variants, suggesting that adaptation to high altitude through the HIF-pathway did not impact CMS genetic architecture. While we confirmed a strong signal of recent positive selection around the genes *SENP1*, *ANP32D* (Zhou et al., 2013), and *PYGM*, *TBX5*, and *SH2B1* (Crawford et al., 2017), we did not replicate selection signals from other studies; this suggests either a differential selection in other Andean populations or potential false positives in reported genes associated with adaptation to high altitude in the Andes (note that our sample size, *N* = 124 for selection scan, outperformed those from previous studies). Overall, our results reveal new insights on the genetic architecture of CMS and do not provide evidence that CMS-associated variants are linked to a strong ongoing adaptation to high altitude, suggesting that Andeans present a functional adaptation to high altitude similar to low-altitude dwellers, rather than high-altitude dwellers from East Africa or Tibet where the prevalence of CMS is low. It might be interesting to note that CMS patients travel less frequently to sea level (**Table 1**). However, it is unlikely that short trips (less than 1 week) to low altitude might prevent the development of CMS. Moreover, decreased mobility might also be a consequence of the disease.

The adaptive correlation between [Hb], arterial oxygen saturation, and high-altitude hypoxia has been widely studied and differs in pattern between native Tibetan, Ethiopian, and Andean populations. Indeed, [Hb] in native Tibetans and Ethiopians responds to high altitude to a lower extent than Andeans (Beall and Reichsman, 1984; Beall, 2006). Tibetans and Ethiopians living at 3,500–4,000 m show [Hb] similar to what is observed in populations living at sea level but with arterial hypoxemia in Tibetans and normoxemia in Ethiopians. [Hb] increases at higher altitudes in Tibetans, highlighting that the hypoxic pathway is still functional. In contrast, in Andean highlanders living at the same altitude, mean hemoglobin concentration is higher and arterial oxygen saturation is reduced compared to sea level populations (Beall and Reichsman, 1984; Beall et al., 1998; Beall, 2006). CMS, characterized by excessive erythrocytosis leading to reduced life expectancy, is quite common in Andean native populations and in lowlanders living at high altitude but is rarely observed in Tibetans. This shows the prominent role of [Hb] in adaptation to high altitude and a better adaptation of Tibetans to altitude than Andeans (Zhang et al., 2017). Interestingly, although mean [Hb] appears highly different between these populations, the heritability of this trait is quite high with 0.86 in Tibetans and 0.87 in Bolivians (Beall et al., 1998). The common finding that 86–87% of [Hb] is attributable to genetic factors in contrast to the large difference in [Hb] among populations suggests that different alleles influence the hematological response to high altitude.

Our study includes both subjects with pathological or normal [Hb] in response to altitude hypoxia, sampled from the same population in one of the highest cities in the world (Cerro de Pasco; 4,380m); this maximizes the chance of discovering the genetic determinants involved. Here, we aimed to decipher the genetic basis underlying the risk of CMS in Andeans and whether this risk is linked to Andean adaptation to altitude. We set up a research protocol based on three non-exclusive hypotheses: i) CMS could be the consequence of an incomplete adaptation to altitude due to shorter exposure to altitude (at the population level) when compared to Tibetans or Ethiopians, ii) the population history of Andeans, with migrations between high and low altitude regions and ancient and recent population admixture, could have reduced adaptation to altitude, iii) and finally, clinical frame of CMS is mainly independent from altitude adaptation. We set up the largest GWAS study on altitude adaption in Andean with 166 CMS patients and 146 healthy subjects. Our genomic analyses of adaptation to high altitude in Andeans resulted in several important observations that shed a new light on the biology and population processes involved that are discussed in detail below.

First, only one SNP, rs7304081, in chromosome 12 close to *AEBP2* reached the genome-wide statistical significance threshold; the ancestral lowlander allele rs7304081-C is overrepresented in CMS patients. *AEBP2* is the only differentially expressed gene in peripheral whole blood between CMS patients and controls. *AEBP2* is an epigenetic regulator for neural crest cells but has never been implicated before in hypoxia/altitude adaptation processes (Kim et al., 2011). At a discovery threshold of 10^−5^, three additional SNPs were detected, rs75810402, rs7832232, and rs7168430. Close to rs75810402 on chromosome 5 is *CAST* and close to rs7168430 on chromosome 15 is *MCTP2*, two candidate genes also suggested using expression data. Calpastatin, the protein encoded by *CAST*, has an inhibiting effect on calpain and has been evoked in the progression of pulmonary hypertension (Wan et al., 2016). *MCTP2* has been associated with bodyfat levels and obesity (Bouchard et al., 2007). At this point, it is important to recall the pathophysiological hypotheses concerning CMS: depressed ventilation, especially during sleep (aggravated by sleep apnea and overweight), severe hypoxemia, increased secretion of erythropoietin, and excessive erythrocytosis (Richalet et al., 2008; Villafuerte and Corante, 2016); CMS is sometimes associated with pulmonary hypertension due to chronic hypoxic remodeling of the pulmonary vasculature (León-Velarde et al., 1994). Interestingly, the differential expression of *CAST* and *MCTP2*, found in the present study, could be linked with two pathological phenotypes, pulmonary hypertension and overweight, both of which associated with CMS, as shown in the present study. Only a prospective study starting early in age would determine if overweight and obesity are cause or consequence of CMS. In fact, a vicious circle may develop with hypoventilation, hypoxemia, polycythemia, CMS, inactivity, and obesity.

Overall, we did not observe any correlation between the strongest signals of association, natural selection, and excess of European admixture (**Figure 4**). Nevertheless, all the risk alleles of our candidate variants have a lower allele frequency than in lowlanders from Lima, suggesting some selective pressure on these variants, but no strong ongoing adaptation as previously hypothesized. We also observed that genes from HIF-pathway were enriched in signals of natural selection but not in CMS-associated variants (**Table S6**), suggesting that adaptation to high altitude through the HIF-pathway did not impact the genetic architecture of CMS. Several studies with conflicting results identified loci under strong positive selection in Andeans (Bigham et al., 2010; Zhou et al., 2013; Foll et al., 2014; Fehren-Schmitz and Georges, 2016; Crawford et al., 2017), and some of these loci (*SENP1*, *ANP32D*, and *PYGM*) appear also under positive selection in our study but remain independent of CMS phenotype. Convergent adaptation between Tibetans and Andeans has been reported by Bigham et al. (Bigham et al., 2010) and Foll et al. (Foll et al., 2014) and particularly highlights the response to hypoxia and the role of *EPAS1* and *EGLN1* in adaptation to altitude. It remains nevertheless striking that *EPAS1* and *EGLN1* have been reported in the literature as modulators of [Hb] only in Tibetans. Broadly, it appears that the physiological mechanisms of adaptation to altitude-induced hypoxemia differ between Tibetans [lowlander [Hb], high blood flow, high resting ventilation and hypoxic ventilatory response (Beall, 2007), and high NOS activity (Erzurum et al., 2007)] and Andeans [high [Hb] and hypoxemia (Beall, 2007)], suggesting that convergent adaptation, based on a functional point of view, does not shape adaptation to hypoxemia in both populations. We failed to show common genome-wide significant association or discovery signals between our Andean CMS GWAS study and the selection studies, including intragenic *EPAS1*- and *EGLN1*-SNPs. Although the largest ever presented for CMS (and high-altitude populations in the Andes), our sample size remains small relative to classical GWAS. Despite this limitation, the power of our study is large enough to detect modulating variants in *EPAS1* and *EGLN1* with the same frequency difference as measured between Tibetan and Han populations (**Figure S4**). These striking results may be due to the low power of our cohort to detect association but may reflect that adaptation to altitude in Andeans is still ongoing, subject to specific population history, or acting mainly on [Hb]. The evidence that [Hb] is higher in non-CMS Andean population when compared to Tibetans living at the same altitude demonstrates that either adaptation is still incomplete or that [Hb] is subject to balanced selection in this population. Alleles increasing [Hb] could be beneficial in youth especially in women of reproductive age living at high altitude, while it could impair fitness in the elderly but with a lower evolutionary cost.

Second, we thus postulated that the population history of Andeans might have influenced the selection hallmark of high altitude on genome. Consistent with the absence of selection on *EPAS1*, the five SNPs of the Denisovan haplotype under selection in Tibetans (Huerta-Sánchez et al., 2014) were absent in our cohorts. Whether this haplotype was initially present in early settlements of Native Americans remains to be established. Another important population admixture situation could have impaired the process of adaptation to high altitude. Native Americans experienced a strong population bottleneck coincident with European immigration in the 16^th^ century. Based on a mitochondrial DNA study, O’Fallon and Fehren-Schmitz (O’Fallon and Fehren-Schmitz, 2011) demonstrated that some 500 years before present, female population effective size was reduced by ∼50%, suggesting that population admixture with lowlander European population may have reduced the process of adaptation to altitude. In fact, we observed a tendency to a higher admixture level in CMS when compared to control subjects. Another important point influencing selection with respect to altitude is the difference in the duration of the selection pressure, 25–30,000 years in Tibetans while only 10,000 years in Andeans. Moreover, most CMS symptoms occur in adult maturity, far after reproductive age (20–25 years old is usually considered as the peak of the reproductive period), and thus may expose CMS to a weak selection pressure. To illustrate the impact of population history in adaptation to hypoxia, a recent paper (Zhang et al., 2018) reported that previous studies about high-altitude adaptation in Tibetans were mainly conducted on the Dbus-Gtsang and Amdo Tibetans living in the Qinghai-Tibet plateau area. A third Tibetan population, the Kham Tibetans also living above 3,000 m, has higher hemoglobin count, and around 20% of the population experiences CMS, close to the proportion found in the Andes. Kham Tibetans are situated in the “ethnic corridor of southwest China” where many Han people moved to Tibet during the Qing Dynasty (1644–1911 CE) with a probable high level of admixture between the two populations (Zhang et al., 2018).

In conclusion, CMS may have various determinants. By performing the largest GWAS of CMS, as well as the largest selection scan in native Andeans, we observed no evidence that CMS is a consequence of an ongoing adaptation to altitude where variants under positive selection have not yet reached their optimum frequency. In addition, although the genes of the HIF pathway are under selection in Andeans, these genes are not enriched in the GWAS signal, which does not sustain our first hypothesis that CMS risk variants are under adaptation. Secondly, the influence of genetic admixture with another population, i.e., low interbreeding with Denisovan and recent admixture with lowlander European genomes, may also have a limited impact on adaptation to altitude and CMS outcome. Finally, the physiological pattern of adaptation to hypoxia between Tibetans and Andeans is quite different. In Tibetans, adaptive pathways limit the increase in hemoglobin level, while in Andeans without CMS, a higher [Hb] is a mechanism of adaptation to counteract tissue hypoxia, as it does in all low-altitude natives exposed to high altitude. This mechanism has no major negative impact during youth, and the evolutionary cost, CMS in the elderly, is limited. Finally, without a strong relationship with natural selection in response to altitude-induced hypoxia, CMS may appear as a polygenic trait, involving other risk factors such as ventilatory defects, pulmonary hypertension, or overweight. Thus, a larger sample size will be necessary to replicate the variants we have identified as being associated with CMS, to investigate selection pressure on these variants, and to investigate the role of European admixture.

## Data Availability

The raw data supporting the conclusions of this manuscript will be made available by the authors, without undue reservation, to any qualified researcher.

## Ethics Statement

The study was approved by the Institutional Ethics Committee of Universidad Peruana Cayetano Heredia. All participants were enrolled in the study after signing an informed consent.

## Author Contributions

All the authors read and approved the manuscript.

## Funding

This study was supported by grants from Laboratory of Excellence GR-Ex, reference ANR-11-LABX-0051. The labex GR-Ex is funded by the program “Investissements d’avenir” of the French National Research Agency, reference ANR-11-IDEX-0005-02. The study was also supported by a Wellcome Trust grant 107544/Z/15/Z to FCV.

## Conflict of Interest Statement

The authors declare that the research was conducted in the absence of any commercial or financial relationships that could be construed as a potential conflict of interest.
