# Supplementary material for: The Genetic Architecture of Chronic Mountain Sickness in Peru
Source: Front Genet. 2019 Jul 30;10:690. doi: 10.3389/fgene.2019.00690 (PMC6682665; doi:10.3389/fgene.2019.00690)
Supplement: Supplementary file 1 [file DataSheet_1.docx]

**Complex patterns of natural selection
into the genetic architecture of Chronic Mountain Sickness**


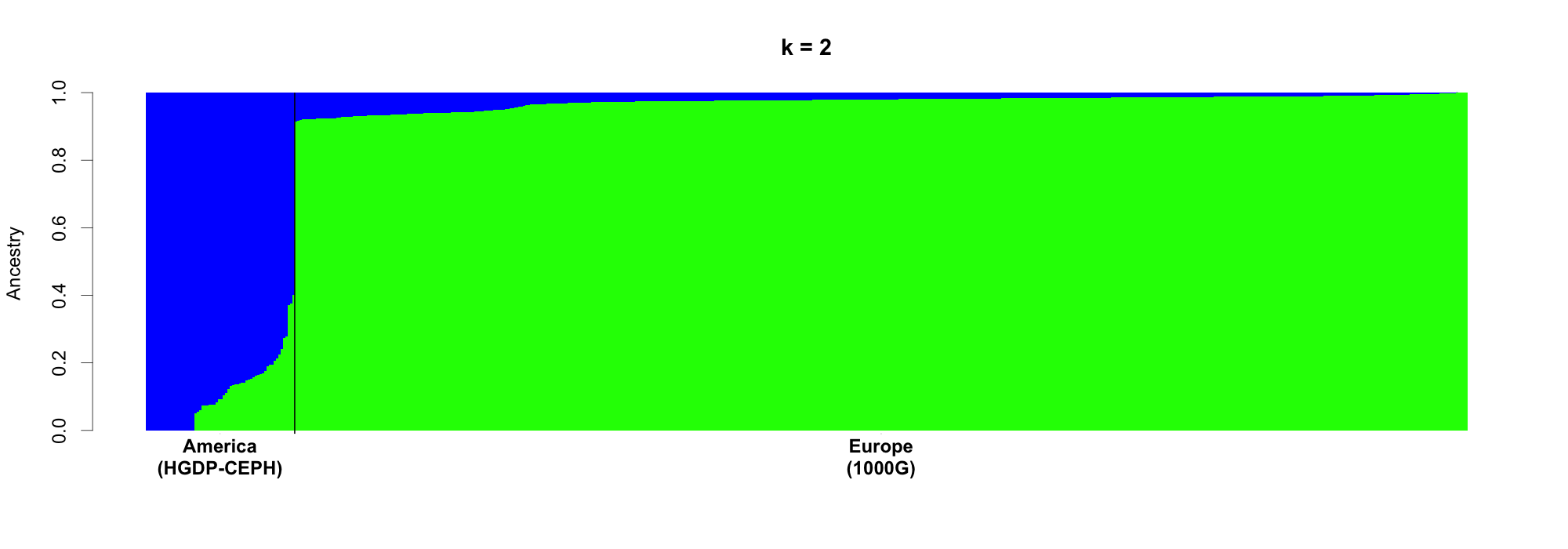


**Figure S1A: Admixture analysis of Native American individuals of HGDP-CEPH panel and European individuals of 1000G panel.** Each vertical line represents an individual. The colors represent the proportion of inferred ancestry from K=2 ancestral population. Admixture analyses of the paper were performed using as reference ancestral populations the 21 American individuals that have been inferred as entirely coming from the fist ancestral population (the 21 individuals of the left, represented by whole blue lines), and the 21 European individuals with the highest proportion of the genome coming from the second ancestral population (the 21 individuals of the right, represented by the longest green lines).

**Figure S1B: European admixture proportions estimated with Admixture and RFMix software.** Admixture software estimates used 21 Native American individuals and 21 European individuals as ancestral populations, while RFMix software estimates used 64 American individuals and 503 European individuals in the reference ancestral haplotype populations. RFMix software estimates were computed by averaging the European admixture proportion estimated at each marker. Grey line represents the equation y = x. The correlation between both estimates is 0.98 (*P* < 10^-10^). The regression coefficient is 0.94 (*P* < 10^-10^) and corresponding intercept is 0.02 (*P* < 10^-10^).

**
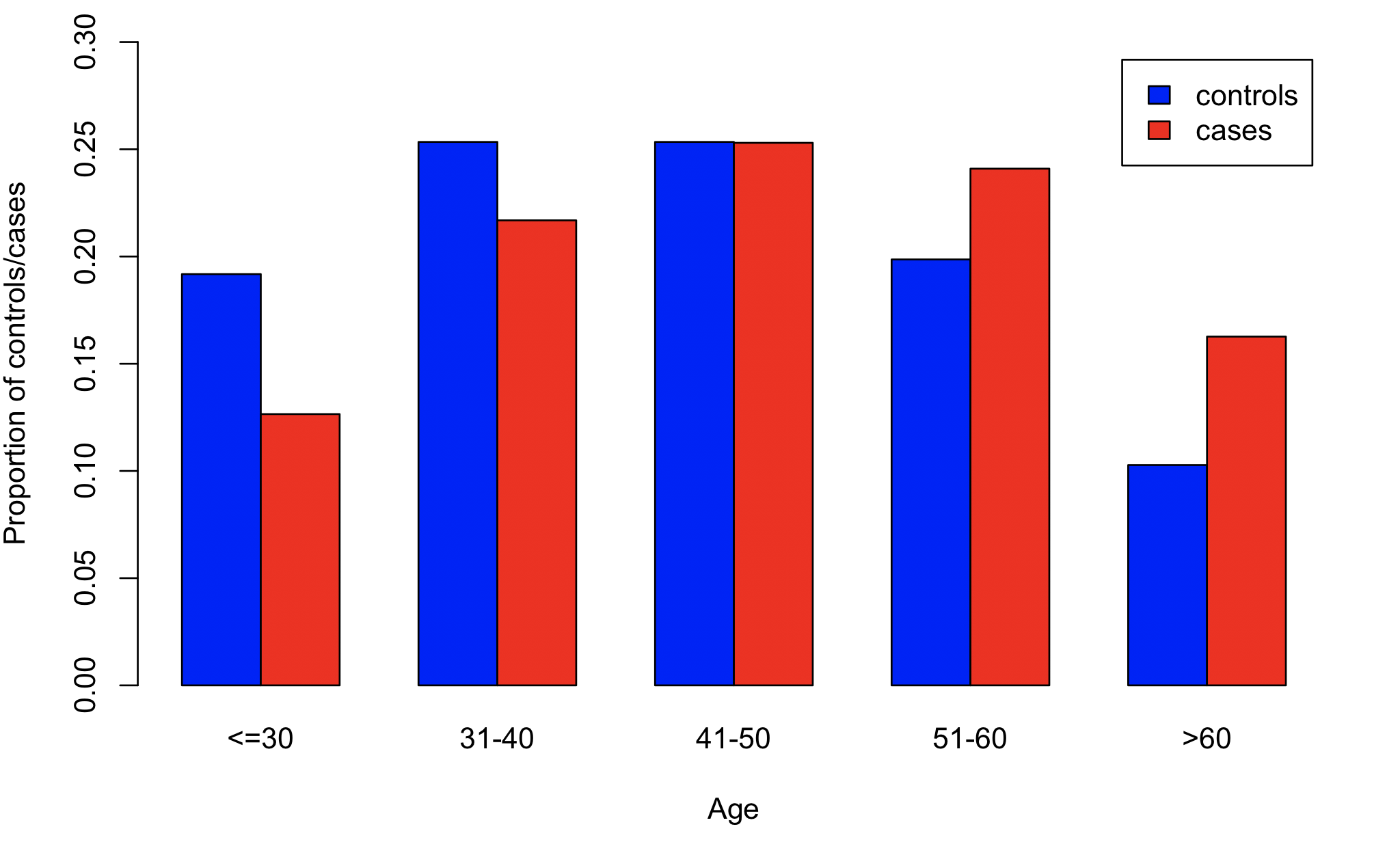
**

**Figure S2. Age distribution in controls and cases.** We observed few cases of CMS below 30 years old, which is the peak of reproductive period.

**
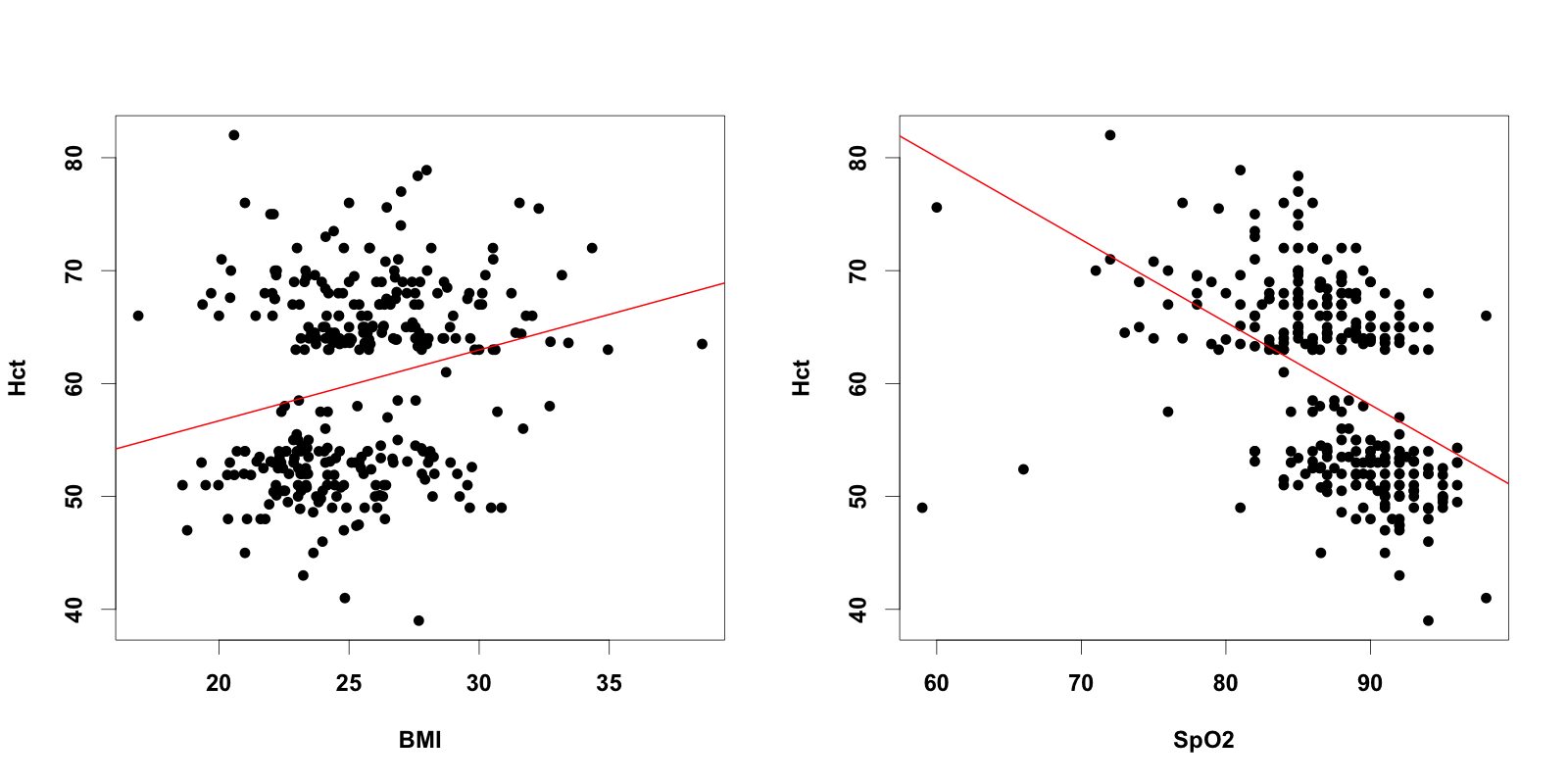
**

**Figure S3. Hematocrit (Hct) as a function of Body Mass Index (BMI) and arterial O_2_ saturation (SpO_2_) in the whole population.** Hct above 60%: CMS patients, Hct under 60%: control subjects. Red lines are regression lines computed on unrelated individuals. Regression coefficient of Hct with BMI = 0.67 (*P* = 6.61 x 10^-5^). Regression coefficient of Hct with SpO_2_ = -0.71 (*P* = 1.19 x 10^-16^).

**Figure S4: Power study with our sample size.** **(a)** We report the minimum genetic effect that we can detect with a power of 95% according to the allele frequency. **(b, c, d)** We report the power of detecting a genetic effect of 2.0 (b), 2.5 (c) and 3.0 (d) according to the allele frequency. We considered significance thresholds of 5 x 10^-8^ (i.e. the genome-wide significance threshold; in black), 1 x 10^-5^ (the candidate threshold used in our GWAS analysis; in red), and 1 x 10^-4^ (the candidate threshold used in our cross results analyses; in grey). We note that our sample size allows to detect modulating variants in *EPAS1* and *EGLN1,* would they show the same frequency difference as measured between Tibetan and Han populations: Yang et al. (2017) observed an odd ratio > 100 for EPAS1 (allele frequency of 0.447 and 0.003 for Tibetan and Han, respectively), and around 10 for EGLN1 (allele frequency of 0.599 and 0.135 for Tibetan and Han, respectively).

**b.**

**Figure S5: Candidate region of chromosome 12.** We report the *P* values across the candidate regions for the genotyped **(a)** and imputed **(b)** SNPs plotted using the LocusZoom software (Pruim et al., 2010) (only SNPs with an rs numbers are displayed, but no informative signal is added when considering all SNPs). The SNP with the lowest *P* value is plotted as a purple diamond; other data points are colored according to their r^2^ in the American population of 1000 Genomes. Grey points indicate missing LD information.

**a.**

**b.**

**Figure S6: Candidate region of chromosome 5.** We report the *P* values across the candidate regions for the genotyped **(a)** and imputed **(b)** SNPs plotted using the LocusZoom software (Pruim et al., 2010) (only SNPs with an rs numbers are displayed, but no informative signal is added when considering all SNPs). The SNP with the lowest *P* value is plotted as a purple diamond; other data points are colored according to their r^2^ in the American population of 1000 Genomes. Grey points indicate missing LD information. (Note that rs200185797 is a deletion, and LD information for this variant is absent from LocusZoom LD database)

**a.**

**b.**

**Figure S7: Candidate region of chromosome 8.** We report the *P* values across the candidate regions for the genotyped **(a)** and imputed **(b)** SNPs plotted using the LocusZoom software (Pruim et al., 2010) (only SNPs with an rs numbers are displayed, but no informative signal is added when considering all SNPs). The SNP with the lowest *P* value is plotted as a purple diamond; other data points are colored according to their r^2^ in the American population of 1000 Genomes. Grey points indicate missing LD information.

**a.**

**b.**

**Figure S8: Candidate region of chromosome 15.** We report the *P* values across the candidate regions for the genotyped **(a)** and imputed **(b)** SNPs plotted using the LocusZoom software (Pruim et al., 2010) (only SNPs with an rs numbers are displayed, but no informative signal is added when considering all SNPs). The SNP with the lowest *P* value is plotted as a purple diamond; other data points are colored according to their r^2^ in the American population of 1000 Genomes. Grey points indicate missing LD information.

**Figure S9: Conditional analyses around each candidate loci.** For each candidate region, we compared the *P* values of the association study (black dots), to the *P* values of an association study conditioning on the locus with the minimum *P* value (grey dots). We did not observe signal independent of the locus with the minimum *P* value.

**Figure S10: Imputed BMI GWAS *P* values around each candidate loci.** For each candidate locus, we compared the *P* values of the CMS association study to the *P* values of the BMI association study in a +/- 1Mb window. We observed that increasing the SNP density does not significantly change the results.

**Figure S11: Imputed RVH GWAS *P* values around each candidate loci.** For each candidate locus, we compared the *P* values of the CMS association study to the *P* values of the RVH association study in a +/- 1Mb window. We observed that increasing the SNP density does not significantly change the results.

**Figure S12: Imputed SpO_2_ GWAS *P* values around each candidate loci.** For each candidate locus, we compared the *P* values of the CMS association study to the *P* values of the SpO_2_ association study in a +/- 1Mb window. We observed that increasing the SNP density does not significantly change the results.

**Figure S13: Gene expression for candidate genes.** *P* values indicate the difference of level of expression in CMS (red) and controls (blue). We only plotted results for genes with a *P* value < 0.05; for other candidate genes see Table S4.

**a.**

**
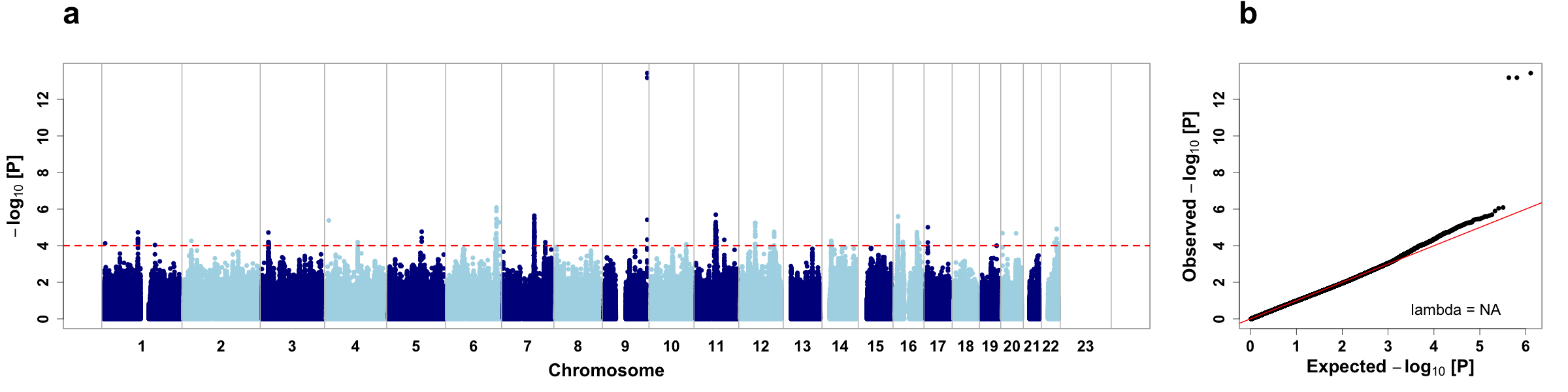
**

**b.**

**
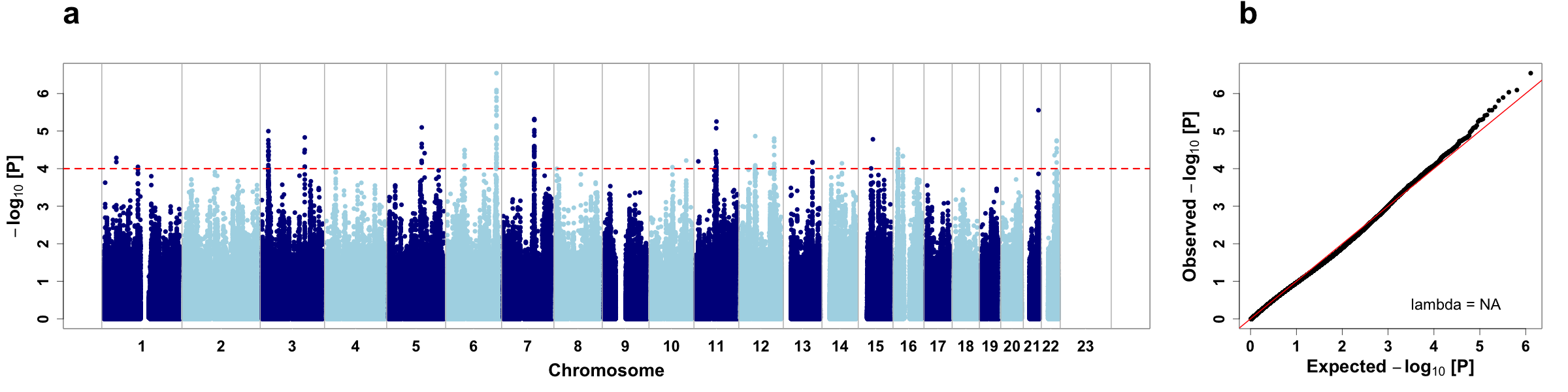
**

**Figure S14: Selection scans performed in 124 unrelated controls.** We report the *P* values of two haplotypic selection tests of recent positive: iHS **(a)** and NSL **(b)**. Results with P < 10^-4^ were consistent across the 2 methods, even if a strong iHS signal in chromosome 9 was not replicated with the NSL method.

**Figure S15: Association and selection signals around candidate SNPs.** We report the GWAS, iHS, nSL and F_ST_ *P* values for 6 candidate SNPs. The 4 first candidate SNPs have been selected through a GWAS *P* value < 10^-5^. The 2 last candidate SNPs have been selected through a GWAS *P* value < 10^-4^ and an F_ST_ P value < 10^-3^. Solid red line represents a *P* value threshold of 10^-5^. Dashed red line represents a *P* value threshold of 10^-3^.

**Figure S16: Association and selection signals around candidate genes.** We report the GWAS, iHS, nSL and F_ST_ *P* values for 21 candidate genes (see Table S1 for references). Solid red line represents a *P* value threshold of 10^-5^. Dashed red line represents a *P* value threshold of 10^-3^.

See supplementary TableS1.txt.gz file

**Table S1: Genome-wide association, selection and admixture results.** We report the GWAS effect sizes (beta) of the minor allele and corresponding standard errors and *P* values, the iHS and nSL statistic *P* values computed in unrelated controls, and the admixture mapping effect sizes (beta) and corresponding standard errors and *P* values for all the 1,288,119 genotyped variants. The A1/A2 column indicates the minor/major alleles.

|  | **Alleles** | **BMI** | | **RVH** | | | **SpO_2_** | |
| --- | --- | --- | --- | --- | --- | --- | --- | --- |
| **Variant** |  | **Beta** | ***P*** | **Beta** | ***P*** | **Beta** | | ***P*** |
| rs75810402 (CAST) | A/G | -0.04 (0.43) | 0.83 | -0.04 (0.06) | 0.38 | 1.66 (0.74) | | 9.15E-03 |
| rs7832232 | G/A | 0.66 (0.26) | 7.78E-03 | 0.08 (0.03) | 2.54E-02 | 0.21 (0.45) | | 0.76 |
| rs7304081 (AEBP2) | A/C | 0.69 (0.25) | 8.86E-03 | 0.05 (0.03) | 0.19 | -0.98 (0.44) | | 2.16E-02 |
| rs7168430 (MCTP2) | G/A | 0.07 (0.28) | 0.95 | 0.02 (0.04) | 0.56 | -1.61 (0.48) | | 3.25E-04 |

|  | **Alleles** | **CMS** | | **CMS adjusted** | |
| --- | --- | --- | --- | --- | --- |
| **Variant** |  | **Beta** | ***P*** | **Beta** | ***P*** |
| rs75810402 (CAST) | A/G | -0.30 (0.07) | 7.37E-06 | -0.25 (0.06) | 1.52E-05 |
| rs7832232 | G/A | 0.19 (0.04) | 2.63E-06 | 0.18 (0.04) | 5.48E-07 |
| rs7304081 (AEBP2) | A/C | 0.23 (0.04) | 4.58E-09 | 0.17 (0.03) | 1.74E-06 |
| rs7168430 (MCTP2) | G/A | 0.19 (0.04) | 6.48E-06 | 0.14 (0.04) | 4.68E-03 |

*CMS adjusted: association with CMS adjusted on BMI, RVH and SpO_2_*

**Table S2: Association of the candidate variants with body mass index (BMI), right ventricular hypertrophy (RVH) and oxygen saturation (SpO_2_).** We report the effect size (beta) of the minor allele and corresponding standard error and *P* value for the different phenotypes using a linear mixed model on 166 CMS patients and 146 controls. The Alleles column indicates the minor/major alleles. The genes between parentheses report the SNP candidate gene based on expression analyses.

| **Variable** | **Coefficient** | ***P*** |
| --- | --- | --- |
| rs75810402 | -1.36 (0.47) | 3.93 x 10^-3^ |
| rs7832232 | 0.90 (0.26) | 6.41 x 10^-4^ |
| rs7304081 | 0.99 (0.26) | 1.22 x 10^-4^ |
| rs7168430 | 0.61 (0.27) | 2.55 x 10^-2^ |
| BMI | 0.17 (0.06) | 7.68 x 10^-3^ |
| SpO_2_ | -0.21 (0.04) | 1.49 x 10^-6^ |
| EUR | 0.36 (2.87) | 0.90 |

**Table S3: Conditional analysis.** We report the logistic regression coefficient and corresponding standard error and *P* value for the four candidate variants, body mass index (BMI), oxygen saturation (SpO_2_) and European admixture proportions (EUR) in a multivariate logistic regression analysis of 143 CMS patients and 124 controls (all unrelated). We note that European admixture proportions (EUR) are still not significant in this analysis.

| **Candidate Loci** | **Gene name** | **Effect size (s.e.)** | ***P* value** |
| --- | --- | --- | --- |
| **rs7304081 (chr 12)** | CAPZA3 | 0.10 (0.12) | 0.417 |
|  | PLEKHA5 | -0.35 (0.55) | 0.524 |
|  | *AEBP2** | *-1.59 (0.77)* | *0.038* |
|  | PDE3A | -0.47 (0.55) | 0.396 |
|  | PYROXD1 | -0.11 (0.65) | 0.871 |
|  | RECQL | -0.17 (0.87) | 0.842 |
|  | SPX | 0.11 (0.44) | 0.802 |
| **rs75810402 (chr 5)** | SLF1 | -0.85 (0.87) | 0.330 |
|  | MCTP1 | -0.43 (0.58) | 0.461 |
|  | ARSK | -0.88 (0.75) | 0.238 |
|  | GPR150 | -0.01 (0.27) | 0.960 |
|  | RFESD | 0.61 (0.41) | 0.136 |
|  | RHOBTB3 | 0.30 (0.57) | 0.603 |
|  | GLRX | -0.24 (0.34) | 0.483 |
|  | ELL2 | 1.09 (1.08) | 0.310 |
|  | *CAST** | *-1.89 (0.86)* | *0.029* |
|  | ERAP1 | -1.00 (0.61) | 0.103 |
|  | ERAP2 | -0.45 (0.37) | 0.216 |
|  | LNPEP | -0.70 (0.70) | 0.314 |
| **rs7832232 (chr 8)** | FGFR1 | -0.62 (0.45) | 0.168 |
| **rs7168430 (chr 15)** | *MCTP2** | *-2.42 (0.81)* | *0.003* |
| **Literature (see Table S4 for references)** | EPAS1 | -0.80 (0.42) | 0.060 |
|  | EGLN1 | -1.37 (0.72) | 0.056 |
|  | EDNRA | 0.06 (0.18) | 0.746 |
|  | *SENP1** | *-2.59 (0.98)* | *0.008* |
|  | ANP32D | 0.11 (0.19) | 0.556 |
|  | PRKAA1 | -0.56 (0.57) | 0.328 |
|  | EDNRB | -0.06 (0.47) | 0.904 |
|  | *ATM** | *-1.45 (0.71)* | *0.041* |
|  | *VEGFA** | *-1.68 (0.73)* | *0.021* |
|  | PDP2 | -0.83 (0.51) | 0.101 |

**Table S4: Association between 36 genes and CMS status.** We report the effect size, and corresponding standard errors and *P* values of gene expression (measured in 71 unrelated CMS patients and controls) association with CMS status. A negative effect size means that decrease expression increases the CMS risk, while a negative effect size means that increase expression increases the CMS risk. Asterisks indicate genes with a significant association with the CMS status at the level 5%. Lines highlighted in grey indicate candidate genes for the candidate loci.

| **Candidate Gene** | **References** | **Population and Analysis** |
| --- | --- | --- |
| **EPAS1** | (Simonson et al. 2010; Yi et al. 2010; Xu et al. 2011; Peng et al. 2011) | Test of selection in Tibet + association with a hypoxia-tolerance phenotype  Genotyping, Tibet (N=46)  Genotyping, Tibet (N=1334) |
| **EGLN1** | (Bigham et al. 2010; Peng et al. 2011; Xu et al. 2011) | Test of selection in Tibet (*N* = 49) and Andes (*N* = 49)  Genotyping, Tibet (N=46)  Genotyping, Tibet (N=1334) |
| **EDNRA** | (Simonson et al. 2010) | Genotyping, Tibet, (N=31) |
| **SENP1 ANP32D** | (Zhou et al. 2013) | Test of selection in Andes + transcriptional response to hypoxia (*N* CMS = 10, *N* non-CMS = 10) |
| **PRKAA1** | (Bigham et al. 2010) | Test of selection in Tibet (*N* = 49) and Andes (*N* = 49) |
| **EDNRB** | (Udpa et al. 2014) | Whole genome sequencing, Ethiopia (N=13) |
| **ATM** | (Appenzeller et al. 2006) | Candidate gene (5) approach (*N* = 30) |
| **VEGFA** | (Buroker et al. 2012; Espinoza et al. 2014) | Candidate gene approach (*N* = 215) |
| **VEGFB** | (Eichstaedt et al. 2014) | Genotyping, Andes (N=50) |
| **PDP2** | (Xing et al. 2008) | Gene expression, Andes (N=20), Ladakh (N=13) |
| **SGK3**  **COPS5 PRDM1**  **IFT122** | (Stobdan et al. 2017) | Test of selection in Andes + down-regulation of orthologs in Drosophila (*N* CMS = 50, *N* non-CMS = 44) |
| **BRINP3**  **NOS2**  **SH2B1**  **TBX5**  **PYGM** | (Crawford et al. 2017) | Test of selection in Andes (*N* = 42) |

**Table S5: List of CMS candidate genes or genes being under adaptation to high altitude (from the literature)**

|  | **Gene +/- 0kb** | | | **Gene +/- 20kb** | | | **Gene +/- 50kb** | | |
| --- | --- | --- | --- | --- | --- | --- | --- | --- | --- |
|  | **OA05** | **OA01** | **FP** | **OA05** | **OA01** | **FP** | **OA05** | **OA01** | **FP** |
| **CMS GWAS** | 0.578 | 0.546 | 0.695 | 0.617 | 0.488 | 0.649 | 0.353 | 0.154 | 0.357 |
| **iHS in controls** | 0.123 | 0.180 | 0.154 | *0.007* | 0.243 | 0.148 | 0.054 | 0.213 | 0.149 |
| **nSL in controls** | *0.003* | *0.004* | *0.022* | *0.002* | *0.021* | *0.009* | *0.009* | *0.002* | *0.004* |

**Table S6: hypoxia-inducible factor (HIF)-pathway analysis.** We report the P values of the HIF-pathway for each study (i.e. CMS GWAS, his and nSL), by retaining the minimal P value inside the gene body (columns Gene +/- 0kb), in a +/-20 kb window around the gene (columns Gene +/- 20kb), and in a +/-50 kb window around the gene (columns Gene +/- 50kb). We computed association enrichment using an over-representation analysis with a significance threshold of 0.05 (OA05), using an over-representation analysis with a significance threshold of 0.01 (OA01), and using the original Fisher product method (FP). Values in italic are below 0.05.
